# The Microbial Assay for Risk Assessment (MARA) in the Assessment of the Antimicrobial Activity of Ofloxacin and Its Photoproducts

**DOI:** 10.3390/ijms26062595

**Published:** 2025-03-13

**Authors:** Grzegorz Nałęcz-Jawecki, Jakub Mielniczek, Milena Wawryniuk, Joanna Giebułtowicz, Agata Drobniewska

**Affiliations:** 1 Department of Toxicology and Food Science, Medical University of Warsaw, 02-091 Warszawa, Polandadrobniewska@wum.edu.pl (A.D.); 2 Department of Drug Chemistry, Pharmaceutical and Biomedical Analysis, Medical University of Warsaw, 02-091 Warszawa, Poland; jgiebultowicz@wum.edu.pl

**Keywords:** photodegradation, MARA, Microtox^®^, Spirotox, fluoroquinolones

## Abstract

Ofloxacin is one of the most commonly used antibacterial substances in the world. Like most medicines, it ends up in the environment through municipal sewage and undergoes various transformations, e.g., photodegradation. The aim of this study was an extensive analysis of ofloxacin photodegradation in both pure antibiotic and a commercial eye drop forms. In this study, a sunlight simulator, chromatographic methods of quantitative and qualitative determination, and biological methods for the evaluation of toxicity (Microbial Assay for Risk Assessment (MARA), Microtox^®^ and Spirotox) were used. The results showed that ofloxacin decomposed almost completely over 2 h of irradiation. Based on the high resolution mass spectrometry, 22 photoproducts were identified. The most sensitive strain of bacteria in the MARA test (*Delftia acidovorans*) responded at a concentration of 7.6 µg L^−1^ of ofloxacin. The antibacterial activity of the irradiated samples was higher than that predicted based on the ofloxacin concentration. This suggests that the resulting photoproducts may have a bacteriostatic effect. The results of additional acute toxicity tests indicate the formation of toxic photoproducts, so it is reasonable to use other organisms that are not focused on a specific target. Such actions may allow for the capture of other, unexpected effects of formed photoproducts.

## 1. Introduction

Ofloxacin (7-fluoro-2-methyl-6-(4-methylpiperazin-1-yl)-10-oxo-4-oxa-1-azatricyclo [7.3.1.0^{5,13}]trideca-5 (13),6,8,11-tetraene-11-carboxylic acid, OFLX) is the second largest-selling fluoroquinolone drug in the US after ciprofloxacin, with 1,963,627 prescriptions in 2022, and the most popular ophthalmic and optic anti-infective with 35% and 67% of the market, respectively [1]. OFLX is on the WHO Essential Medicines List in the ophthalmological preparations section [2]. As a second-generation fluoroquinolone, OFLX has a broad spectrum of activity not only against Gram-negative but also Gram-positive bacteria [3,4,5]. Therefore, it is indicated in many different bacterial infections. OFLX is mostly used as eye and ear drops; however, the oral form of application is also registered and practiced [5]. OFLX is completely eliminated by the kidneys [5] and, like most medicines, it ends up in the environment through municipal sewage. OFLX has been detected in hospital wastewater in Spain at concentrations of up to 4.8 µg L^−1^ [6] and in surface water in China at concentrations of up to 11.7 µg L^−1^ [7]. At such concentrations, OFLX can inhibit the growth of sensitive microorganisms, including cyanobacteria (blue-green algae). Ferrari et al. [8] reported that in a chronic test with the blue-green alga *Synechococcus leopolensis*, the effective concentration (NOEC) of OFLX was 5 µg L^−1^. A much more dangerous effect of the presence of antibiotics in the aquatic environment is the possibility of inducing microbial resistance to antibiotics, which can be caused by sub-microgram concentrations of antibiotics [9,10]. This resistance can be induced not only by a specific antibiotic but also by substances with the same mechanism of action. Furthermore, in the environment, all active compounds undergo numerous abiotic and biotic transformations, e.g., photodegradation, hydrolysis, accumulation in sediments, and biodegradation. The most significant form of abiotic transformation of pharmaceuticals in ground water is photolysis [11].

Fluoroquinolones vary in their sensitivity to UV–Vis light-induced photodegradation. Hubicka et al. [12], examining the effect of UVA radiation on four fluoroquinolones, found that their decomposition occurs according to first-order kinetics, and the rate of photodegradation increases in the following order: norfloxacin < ciprofloxacin < OFLX < moxifloxacin. Ninety percent degradation of danofloxacin, ciprofloxacin, and enrofloxacin was achieved in less than 30 min of full sunlight exposure [13], while the same percentage degradation required 120 and 240 min of exposure for levofloxacin (S-enantiomer of OFLX) and moxifloxacin. OFLX was stable during 90 min of irradiation with visible light [14,15]. Frąckowiak et al. [16] found that OFLX was completely degraded within 150 min of irradiation with UVC radiation (210 nm). Photodegradation rarely leads to complete decomposition of the substance. In the case of OFLX, several products were identified during photolysis and/or photocatalysis, most often resulting from either direct or ring-opening hydroxylation [14,15,17]. The resulting products may exhibit biological activity against both prokaryotic (bacteria) and eukaryotic organisms. The toxicity of compounds formed during oxidation/photolysis processes is often determined only in silico, using quantitative structure–activity relationship (QSAR) predictions based on standard organisms used in ecotoxicology, e.g., alga, daphnia, and fish [18,19]. However, fluoroquinolones are bacteriostatic antimicrobials and exert their activity through binding to bacterial topoisomerase and DNA gyrase [20]. Thus, the toxicity of fluoroquinolones and active antibacterial derivatives should be tested using chronic tests on bacteria or cyanobacteria. Moreover, QSAR analyses indicate only the potential toxicity of individual substances, while the toxicity of the post-reaction mixture may be different due to interactions between the components in the mixture.

The microbial assay for risk assessment (MARA) was developed in 2003 [21]. It enables simultaneous evaluation of the chronic toxicity of a sample for ten bacterial strains and one yeast strain. Due to the different sensitivity of the microorganisms used in the test, in addition to the microbial toxic concentrations (MTCs) for individual microorganisms, the MARA test produces a so-called “toxic fingerprint”, which is an individual toxicity pattern of the tested sample. This makes it possible to observe changes in the spectrum of the toxic effect of the sample, e.g., during photodegradation. The MARA test has been used to assess the toxicity of various substances, including antibiotics [22] and 3,5–dichlorophenol [23], as well as the efficiency of the degradation of environmental pollutants [24,25]. In contrast to the MARA system, acute toxicity tests are used to assess the toxicity of substances that act non-specifically. The most commonly used test for this purpose is the luminescent bacteria test with *Aliivibrio fischeri* (previously known as *Vibrio fischeri* or *Photobacterium phosphoreum*), which allows for the detection of toxic substances that disrupt bacterial metabolism. The results of the luminescent bacteria test allow for the prediction of the toxicity of low-molecular-weight compounds to higher organisms, including *Daphnia* spp. and fish. Such chemical compounds do not have a specific mechanism of action, unlike macromolecular compounds which may be, e.g., photoproducts. A better tool for assessing and predicting the toxicity of photoproducts may be tests using simple eukaryotic organisms, such as protozoa *Spirostomum ambiguum* (Spirotox).

The aim of this study was to comprehensively evaluate the photodegradation of OFLX using a SunTest CPS+ sunlight simulator. To evaluate the effect of formulation on this process, both pure antibiotic and commercial eye drops were tested. OFLX concentration was assessed using HPLC with a diode array detector (HPLC-DAD), while the formed products were detected using UHPLC with a high-resolution mass spectrometer (HR MS/MS). Three bioassays were applied for evaluation of toxicity: MARA—a chronic toxicity assay with 10 species of bacteria and 1 species of yeast [23]; Microtox^®^—an acute toxicity assay with the luminescent bacteria *A. fischeri*; and Spirotox—an acute toxicity assay with protozoan *S. ambiguum* [26]. The MARA test was used to assess the antibacterial activity of the photoproducts formed during the process. The Microtox^®^ and Spirotox tests were used to assess whether the process did not produce products that were acutely toxic to other organisms.

## 2. Results

OFLX, both in pure solution and in the form of Floxal eye drops, was irradiated in the SunTest CPS+ sunlight simulator. The toxicity of unirradiated solutions and solutions irradiated for 30, 45, 60, 90, and 120 min was assessed using the MARA, Spirotox, and Microtox^®^ tests. Toxicity units were calculated for the most sensitive bacteria and compared with the toxicity predicted from OFLX concentrations measured during irradiation. Simultaneously, the concentration of OFLX was determined in all samples, and an attempt was made to identify the formed photoproducts.

### 2.1. OFLX and Floxal Toxicity

The MARA test offers a unique opportunity to simultaneously assess the chronic toxicity of 11 strains of microorganisms. Table 1 presents the MTC values for the tested strains, starting from the most sensitive one. The MTC values were calculated on the basis of the OFLX concentration in samples and expressed in µg L^−1^. OFLX inhibited the growth of *D. acidovorans* bacteria at concentrations of 7.6 to 21.2 µg L^−1^, with Floxal being on average two times less toxic. The next two strains of Gram-negative bacteria, *Citrobacter freudii* and *Comamonas testosterone*, were 5.2 to 8.0 times less sensitive than *D. acidovorans*, although their MTC values below 100 µg L^−1^ also indicate the high sensitivity of these bacterial strains to this antibiotic. In contrast, the Gram-positive bacteria *Kurthia gibsonii*, *Microbacterium* sp., and *Staphylococcus warneri* and Gram-negative *Pseudomonas aurantiaca* were 30 to 65 times less sensitive than *D. acidovorans*, with average MTC values between 470 and 1000 µg L^−1^. The remaining bacterial strains and *Pichia anomala* yeasts did not respond to the tested antibiotic even at the highest tested concentrations of 1500 µg L^−1^. The sensitivity of bacteria to the Floxal eye drops was comparable to that of the pure antibiotic OFLX, with the exception of *D. acidovarans*, which had two times lower sensitivity, and the slightly lower sensitivity of *C. testosterone* and *P. aurantiaca*.

The luminescent bacteria *A. fischeri* and the protozoa *S. ambiguum* used in the Microtox^®^ and Spirotox assay, respectively, did not respond even to the highest OFLX concentration used, that of 30 mg L^−1^. The obtained results indicate the absence of any harmful substances in the tested samples other than the bacteriostatic antibiotic OFLX.

### 2.2. The Concentration of OFLX During Irradiation

**Figure 1:**
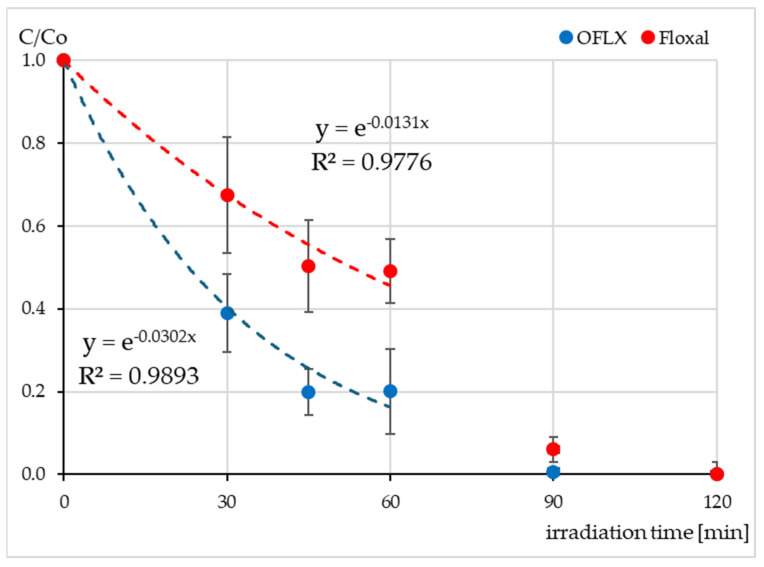
Relative concentration of OFLX during photodegradation of the standard OFLX solution (•) and Floxal eye drops (•) in the SunTest CPS+. The initial OFLX concentration Co was 30 mg L^−1^.

The OFLX solution and Floxal eye drops were irradiated in the SunTest CPS+ sunlight simulator for 120 min. The concentration of the OFLX was determined with the HPLC-DAD. The concentration of OFLX, both in the standard solution and Floxal, decreased gradually until 60 min of irradiation and then rapidly dropped to a few percent of the initial value (Figure 1). The OFLX concentration was below the limit of quantitation (0.05 mg L^−1^) after 120 min irradiation in both samples. The degradation of OFLX in the standard solution was more than two times faster than in the eye drop formulation, and its t½ was 23 min compared to 53 min.

### 2.3. The Effect of Irradiation on the OFLX and Floxal Toxicity in the MARA Assay

**Figure 2:**
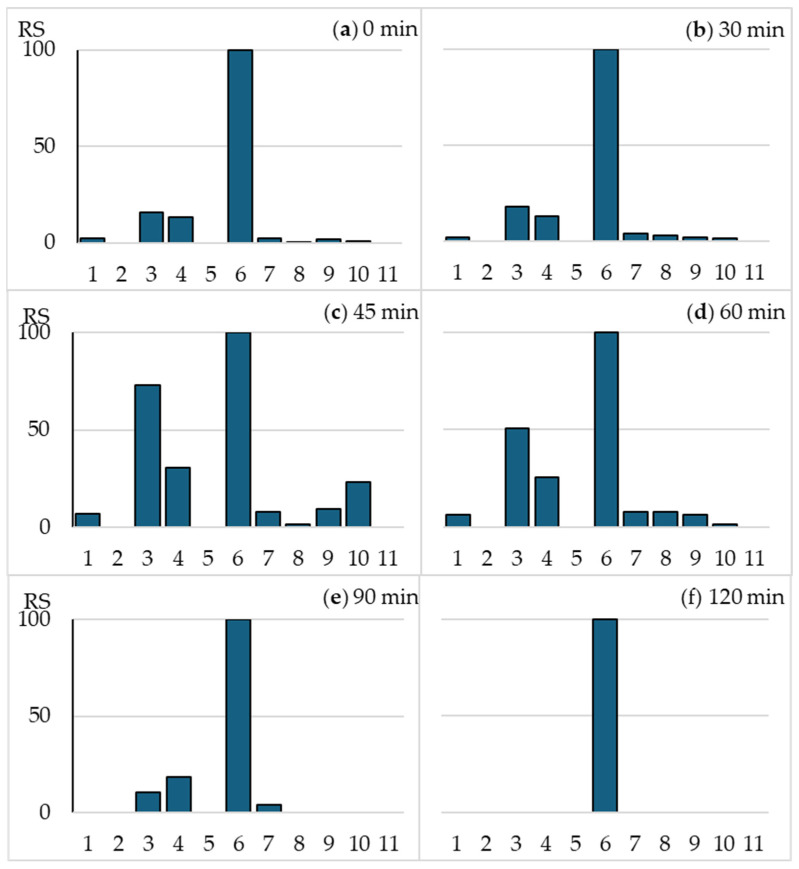
The sensitivity of MARA assay during photodegradation of OFLX and relative sensitivity (RS) calculated for each strain in comparison with the most sensitive strain, No 6 (RS = 100). Irradiation time: (**a**) 0 min; (**b**) 30 min; (**c**) 45 min; (**d**) 60 min; (**e**) 90 min, and (**f**) 120 min.

The relative sensitivity (RS) of all 11 strains used in the MARA test, defined as the fingerprint, was calculated using Equation (1) (Section 4.5). The RS did not change during the first hour of irradiation of the samples (Figure 2). The gradual decrease in OFLX toxicity towards strain No. 6 after 45 and 60 min resulted only in a relative increase in the RS values of the remaining strains. After 90 min of exposure, antibacterial activity decreased significantly; the sample inhibited the growth of only four bacteria, while after 120 min, a slight growth inhibition was noted only for the most sensitive strain, No. 6 (Figure 2e,f).

**Figure 3:**
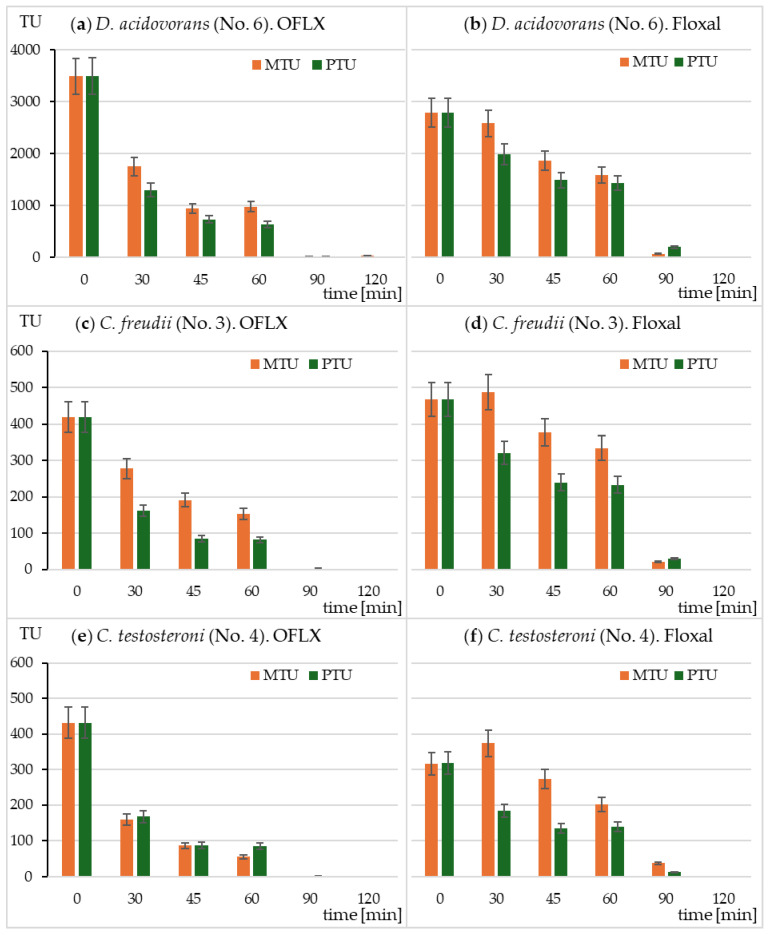
Measured (MTU) and predicted (PTU) toxicity units during photodegradation of OFLX and Floxal samples in the SunTest CPS+. The PTU values were calculated based on the OFLX concentration in the sample. Results of toxicity to *D. acidovorans* (strain No 6) (**a**) OFLX; (**b**) Floxal; *C. freudii* (strain No 3) (**c**) OFLX; (**d**) Floxal; and *C. testosteroni* (strain No 4) (**e**) OFLX; (**f**) Floxal.

Based on the OFLX concentrations determined during irradiation and the MTC values for the individual strains, the predicted toxicity units (PTUs) were calculated using Equation (2) (Section 4.8). Since only three strains were sensitive to the samples for up to 90 min of irradiation, PTU values were calculated only for *D. acidovorans*, *C. freudii*, and *C. testosteroni*. For the same strains, the values of measured toxicity units (MTUs) were calculated using the Equation (3) (Section 4.8). The comparison of PTU and MTU values for both tested solutions is presented in Figure 3. The PTU value indicates the contribution of OFLX to the total toxicity of the sample. In the case of the most sensitive bacterium, *D. acidovorans*, the OFLX concentration values well predicted the toxicity of the samples, both in the standard and Floxal drug solutions (Figure 3a,b). However, in the case of *C. freudii* (both solutions) as well as *C. testosteroni* (Floxal), the toxicity caused by OFLX accounted for only a part of the total toxicity of the samples, especially after irradiation times of 30 and 45 min (Figure 3c,d,f). These results indicate the formation of toxic photoproducts for the above organisms with slightly different spectra of activity. The differences between MTU and PTU values decreased with time, which shows the photolability of the formed photoproducts.

### 2.4. The Effect of Irradiation on OFLX and Floxal Toxicity in Microtox^®^ and Spirotox Assays

The toxicity of irradiated OFLX and Floxal samples tested with Microtox^®^ and Spirotox assays is presented in Table 2. The EC_50_ values are expressed as a percentage of the highest sample concentration (100% = 30 mg L^−1^). The toxicity of OFLX and Floxal eye drops increased with irradiation time, hence the photoproducts formed during irradiation were more toxic than OFLX. Luminescent bacteria reacted only to photoproducts formed after 90 min and 120 min of irradiation. Their reaction was lower than that of protozoa, and the minimum EC_50_ value was 65%. In this case, Floxal photoproducts were slightly more toxic than those of OFLX. In contrast, protozoan *S. ambiguum* was affected by all irradiated samples. The EC_50_ values decreased from 70% to less than 20% after 30 min and 120 min of exposure, with OFLX showing slightly higher toxicity.

### 2.5. Analysis of Photodegradation Products with UPLC-MS/MS

**Figure 4:**
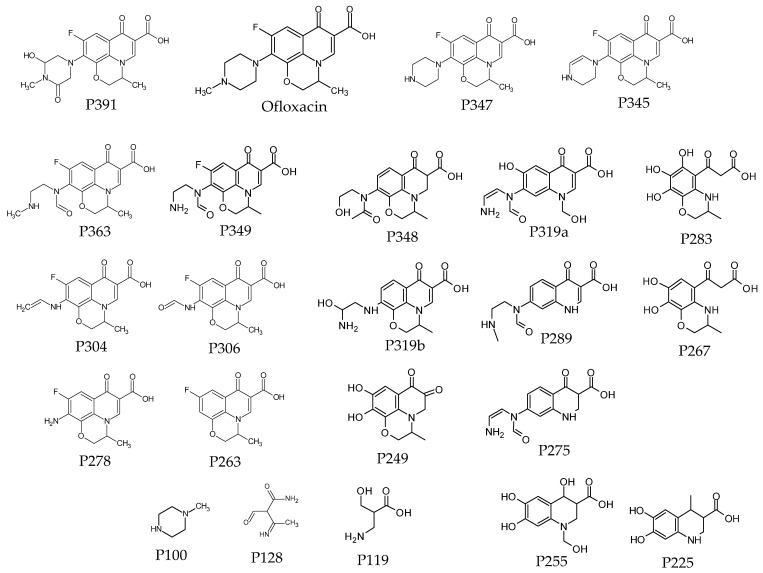
Plausible structure of the OFLX photodegradation products.

UHPLC coupled with the high-resolution MS/MS was used to identify the compounds formed during the OFLX photodegradation. Table 3 presents the substances for which the peak area after irradiation increased at least 10-fold compared to unirradiated samples. Based on the results of UHPLC with high-resolution MS/MS and the prior literature [12,14,15,16,17,19,27,28], 22 plausible OFLX degradation intermediates were proposed (Table 3, Figure 4). The irradiated OFLX underwent various changes. P347 was formed by the cleavage of the methyl group from the piperazinyl ring, while P391 was formed by the oxidation of this ring. The largest number of different derivatives were formed by oxidative decomposition of the piperazine ring (P363, P349, P348, 319b, P306 and P304) and/or the oxazine ring (P319a, P289, P275 and P255). These transformations led to the complete degradation of the rings piperazine (P278 and P263) and oxazine (P225). In more than half of the derivatives (right side of Figure 4), ring degradation was accompanied by cleavage or oxidative cleavage of the fluorine atom. Ultimately, due to all the reactions, the intermediates were decomposed into small molecules (P128, P119 and P100). The obtained results suggest that the products in which the piperazine ring has decomposed may retain their antibacterial activity.

Due to the fact that UHPLC MS/MS analysis was performed at each time point, it was possible to track the time at which the individual intermediates appeared (Figures S1 and S2). The earliest to appear in OFLX solution was P374 (desmethylofloxacin) (Figure S1d). In Floxal solution, this reaction occurred later. However, after 90 min (OFLX) and 120 min (Floxal), this derivative was no longer observed. The largest number of derivatives with the highest abundance were detected after 90 min of irradiation. The compounds with the lowest molar masses, P119 and P128, were observed at the end, after 120 min.

In most cases, the same products were formed in both OFLX solutions and Floxal eye drops after the same exposure time (Figures S1 and S2). The exceptions were P363, P345, and P100, which were formed in significantly larger amounts in Floxal after 90 min, and P319b, P275, and P119, which were practically absent in the irradiated Floxal solution.

## 3. Discussion

In this study, the photodegradation of OFLX was comprehensively assessed using biological (biotests) and chromatographic analysis. For the first time, the photodegradation of OFLX solutions prepared in a standard minimal mineral medium (Tyrode’s medium) and the formulation of Floxal eye drops was compared.

### 3.1. OFLX’s Antibacterial Activity

Fluoroquinolones are broad-spectrum bacteriostatic antibiotics. Their primary mechanism of action involves inhibition of protein synthesis through interaction with gyrase and topoisomerase IV [5]. OFLX is a second-generation fluoroquinolone widely used worldwide. OFLX and its S-enantiomer levofloxacin are among the most widely studied fluoroquinolones [7,29]. Their high concentration in surface waters of up to 11.7 µg L^−1^ [7], combined with high toxicity to cyanobacteria NOEC = 5 µg L^−1^ [8], means that in more than 20 publications, their hazard quotient (HQ) for the aquatic environment exceeds 1 [27]. This means that they pose a high risk to the environment.

OFLX has a strong antibacterial effect, especially against Gram-negative bacteria [4,5]. OFLX at concentrations below 1 mg L^−1^ inhibited the growth of 7 of the 10 bacterial strains used in the MARA test. Three strains were particularly sensitive—*D. acidovorans*, *C. freudii* and *C. testosterone*—for which the mean MTC values were 12.2, 75.6, and 92.5 µg L^−1^, respectively. The same three strains of Gram-negative bacteria were most sensitive to the other fluoroquinolones: ciprofloxacin, norfloxacin, pefloxacin, and flerofloxacin [22]. Also noteworthy is the relatively high bacteriostatic activity of OFLX against two strains of Gram-positive bacteria used in the MARA test, *K. gibsoni* and *Microbacterium* sp., with MTC values around 500 µg L^−1^. OFLX, due to its bacteriostatic activity, is not toxic to luminescent bacteria. Our results confirm previous reports by Bergheim et al. [30], who reported no toxicity of 6 mg L^−1^ levofloxacin in a short-term bioluminescence test with *V. fischeri*.

The slightly lower toxicity of Floxal eye drops compared to the standard antibiotic solution in the MARA assay may be due to the different proportions of isomers in the samples. OFLX is a mixture of two isomers. The S-enantiomer, sold as levofloxacin, is several times more active than racemic OFLX [31]. The HPLC analytical methodology used in the present study does not enable the separation of OFLX isomers, and the presented results indicate the sum of their concentrations. As OFLX in the standard solution and in the Floxal eye drops were derived from different sources, their isomer composition may be different, which may result in different antibacterial activity.

### 3.2. Photodegradation of OFLX

Analysis of the changes of the tested compound during irradiation can be carried out by two methods. Analysis of the sample’s UV–Vis absorption spectrum allows for quick monitoring of changes [32,33]. The intensity of the peaks characteristic of the tested compound allows for estimation of changes in its concentration. Moreover, based on the changes in the spectrum, it is possible to estimate changes in the chromophore of the molecule [33]. In the case of OFLX, the peak at 288 nm attributed to the quinolone substituent is used to analyze antibiotic concentration, while the peak at 226 nm corresponds to the piperazinyl group, and the peak at 330 nm corresponds to the oxazinyl group [34]. However, in order to observe the formation of products in which no changes in the structure of the chromophores occurred, it is necessary to use chromatographic methods enabling the separation of each photoproduct [32]. It is then possible to observe derivatives, e.g., desmethyl ones, whose UV–Vis spectrum is almost identical to the spectrum of the parent compound.

The photodecomposition of OFLX depends on the wavelength of the irradiation light. Since OFLX does not absorb light with a wavelength above 400 nm [3], it does not degrade in visible light [14,15]. Thus, it requires the use of appropriate photocatalysts and/or application of full sunlight, i.e., Vis and UV. In our studies, 80% of OFLX was decomposed during 60 min of exposure in the SunTest CPS+ sunlight simulator. A much slower decomposition was observed by Lu et al. [27], who used monochromatic LED light with a wavelength of 365 nm. The reason for this could be the significantly lower radiation intensity of the LED diode compared to the xenon lamp used in this work. The irradiance in the SunTest CPS+ device is in the range of up to 65 W m^−2^ (300–400 nm) and 750 W m^−2^ (300–800 nm), i.e., it corresponds to the irradiance of sunlight. The degradation of levofloxacin in sunlight was as rapid as in our experiment, with a t½ of about 30 min [34]. However, in the case of OFLX from Floxal eye drops, a slower degradation was observed during the first hour of irradiation, followed by a significant acceleration in the next hour. After 90 min, OFLX was almost completely photodegraded in both samples. Floxal eye drops, in addition to 3 mg mL^−1^ OFLX, contain isotonic saline solution (0.9% NaCl) and benzalkonium chloride (25 µg mL^−1^). Therefore, the concentration of benzalkonium chloride in the tested solution was over 100 times lower than OFLX. The possible influence of benzalkonium salts on the photodegradation process has not been evaluated in the literature. This influence should be determined in future studies.

The degradation of OFLX under the influence of radiation of different wavelengths has been analyzed both without and with the use of photocatalysts [14,15,17,18,19,34]. The photo-reactivity of fluoroquinolones arises from the excitation of the quinolone chromophore after absorption of a photon [27]. Ding et al. [28], using different quenchers, found that the photocatalytic degradation of OFLX with graphene oxide-supported titania/zirconia ternary nanocomposites was mainly due to O_2_^•−•^ radicals, while HO^•^ played only a minor role. Dhiman et al. [14], using a different photocatalyst, also indicated the primary importance of radicals, with a lesser role for holes.

The photodecomposition of fluoroquinolones by direct irradiation has previously been examined [14,15,17,18,19,34]. Several pathways for the transformation of OFLX under irradiation have been proposed, including (1) hydroxylation; (2) hydroxylation with opening of the oxazine ring; (3) cleavage of the piperazine ring; (4) defluorination [17,34]; and (5) demethylation [19,27]. Analysis of irradiated samples using UHPLC with high-resolution MS/MS revealed the presence of 22 substances whose peak areas were more than 10 times larger than those of unirradiated samples (Table 3, Figure 4). The identified OFLX photoproducts agreed well with previous studies on the photodegradation of OFLX [12,14,15,16,17,18,19,27]. Similar to Frąckowiak [16] and Li [17]’s research, derivatives resulting mainly from the decomposition of the piperazine and oxazine ring were observed. The amine-N atom in the piperazine ring is readily oxidized, giving rise to a series of oxygenated (P391 [27]) and ring-opening intermediates (P363, P306, and P304 [19], and P278 [18,19,27]) and finally the cleavage of the entire piperazine ring (P263 [19]). Just as in the studies by Lu et al. [27], Li [17], and Xing et al. [19], defluorination and decarboxylation were also observed.

The novelty of our research lies in the analysis of products after different photodegradation times. Due to the lack of analytical standards, it was not possible to assess their concentrations. However, based on the size of the areas under the peak, it was possible to estimate trends in changes over time. Derivatives that appeared both immediately after 30 min of irradiation (P347 and P319a) and at the end of the process (P255 and P249) were observed. In several cases, significant differences in the abundance of some intermediates were observed, with OFLX in Floxal demonstrating a stronger decomposition of the piperazine ring (P363 and P345), while OFLX in pure solution defluorinated more strongly (P319b and P275). The different rates of formation of various derivatives observed for OFLX solution and Floxal eye drops may be for two reasons. First, different proportions of isomers degraded by different transformation pathways. Second, benzalkonium chloride was present in the drug formulation, the effect of which on the photodegradation of fluoroquinolones is not known.

### 3.3. Toxicity of OFLX Photodegradation Products

The key elements of the molecule [4] for the bacteriostatic action of fluoroquinolones are the N1 position substituted with a small hydrophobic group and 3-carboxyl and 4-carbonyl groups. The fluorine atom in position 6 determines the high potency of the antibacterial action. On the other hand, the substituent in position 7 interacts with DNA gyrase and topoisomerase IV, and its type determines the strength of the interaction with different bacterial strains.

Fluoroquinolones constitute a very large and constantly expanding group of antibiotics [4,20]. However, little is known about the toxicity and antibacterial activity of their transformation products. Desmethyl ofloxacin (also known as ofloxacin impurity E) has moderate antibacterial activity, while ofloxacin N-oxide has only minimal activity [3]. The degradation products P391, P363, P349, P348, P347, P345, P306, and P304 identified in this study retained the basic elements that determine the antibacterial activity listed above. In their case, the changes under the influence of light were mainly related to the piperazine substituent. Hence, these transformation products may retain partial antibacterial activity and may be responsible for the greater activity than OFLX activity against bacteria in the MARA test, which was observed during the irradiation of the samples (Figure 3). The toxicity of the photodegradation products was determined indirectly by comparing the MTU values with the PTU values, which were calculated from the OFLX concentration in the sample. Bergheim et al. [28] found that the toxicity of levofloxacin samples in the chronic toxicity test on *Pseudomonas putida* decreased during irradiation, but more slowly than the antibiotic content. Zhang et al. [35], studying the biotransformation process of ciprofloxacin, observed transformations similar to the photodegradation process: hydroxylation, decarboxylation, and decomposition of the piperazine ring. They observed high antibacterial activity against *Escherichia coli* and *Bacillus subtilis* of both the antibiotic and its transformation products.

In our studies, non-irradiated samples were non-toxic upon acute toxicity *A. fischeri* and *S. ambiguum* tests, while irradiated samples became increasingly toxic. However, this toxicity was minor compared to the bacteriostatic activity. Both the unirradiated and irradiated fluoroquinolone samples were not toxic in the *A. fischeri* luminescent bacteria acute toxicity test and also in relation to human Hep-G2 and/or HeLa cells [30]. Due to a lack of standards, toxicity analysis of individual derivatives was not possible. Ecotoxicity is predicted using in silico methods. Li [17], using T.E.S.T. v.5.1.2 software (T.E.S.T., Washington DC, USA), observed lower toxicity of all OFLX degradation products to Fathead minnow fish. Additionally, in silico analysis using the ECOSAR program showed the 100 times higher acute toxicity to *Daphnia* of ciprofloxacin piperazine ring cleavage products compared to the parent antibiotic [35].

Zgadzaj et al. [36] indicated the potential mutagenic effect of the photochemical transformation products of fluoroquinolones. In silico results also indicate the mutagenic potential of many fluoroquinolone photodegradation products [17,28]. Since increased mutation rates may cause changes leading to antibiotic resistance in bacteria, future studies on the photodegradation of fluoroquinolones should include mutagenicity assessment tests. This will allow confirmation of which functional groups/systems are characterized by their high potential to induce mutagenicity.

## 4. Materials and Methods

### 4.1. Chemicals

The OFLX hydrochloride standard (purity > 99%) was obtained from Merck (Darmstadt, Germany). Moreover, 0.3% eye drops containing 3 mg L^−1^ OFLX were purchased from a pharmacy (Floxal, Dr. Gerhard Mann Chem.-pharm. Fabrik GmbH, Berlin, Germany). According to the manufacturer, the eye drops contain 0.9% NaCl and 0.025 mg L^−1^ of benzalconium chloride as an excipient. The OFLX stock solution was prepared by dissolving 10.0 mg of OFLX standard in 10 mL of 50% methanol (1.0 mg OFLX mL^−1^). The 30 mg L^−1^ working solutions were prepared just before conducting the tests by diluting the stock solutions with Tyrode’s medium, 33 and 100 times in the case of the standard OFLX solution and the eye drops, respectively. Tyrode’s medium contains a minimal amount of inorganic components that allow the protozoan *S. ambiguum* to survive for up to 8 days [37] and is tolerated by other test organisms. It comprises 125 mg NaCl, 3.1 mg KCl, 3.1 mg CaCl_2_, 1.55 mg MgCl_2_, 15.6 mg NaHCO_3_, and 0.78 mg NaH_2_PO_4_ per liter of deionized water.

### 4.2. Photodegradation Experiment

The irradiation process was performed using SunTest CPS+ apparatus (Klimatest, Warsaw, Poland) with a 1500 W xenon lamp providing full-spectrum light (UV–Vis) similar to the spectrum of sunlight. For the photodegradation experiments, the working OFLX solutions and the control (Tyrode’s medium) were transferred into 60 mL quartz tubes. The samples were irradiated in a temperature-controlled chamber for 2 h. The fluence rate was set to 750 W m^−2^, which corresponds to a dose of 2700 kJ m^−2^ h^−1^. The temperature during the experiments was 30–35 °C. The subsamples were taken after 0, 30, 45, 60, 90, and 120 min of irradiation. The test was repeated twice, both for the OFLX standard and Floxal.

### 4.3. Liquid Chromatography with Photodiode Array Detector

The concentration of OFLX was measured using a Shimadzu HPLC instrument equipped with an SPD-M10A photodiode-array detector (PDA). In the gradient analysis used, phase A consisted of a 0.05% aqueous solution of trifluoroacetic acid, and phase B was an acetonitrile. The HPLC-grade acetonitrile and the trifluoroacetic acid were purchased from Merck (Darmstadt, Germany), while deionized water was procured by using a Milli-Q^®^ Direct water purification system (Merck, Darmstadt, Germany). The separation process was carried out on a LichroCART 50 × 4 Purospher STAR RP-18 (3 µm) analytical column (Merck, Darmstadt, Germany). The flow rate was 0.8 mL min^−1^, and the concentration of phase B during the analysis changed according to the following scheme: 0 min: 15%; 1 min: 20%; 5 min: 90%; 6 min: 90%; and 6.10 min: 15%. Quantitative analysis of OFLX was carried out at 294 nm. A standard curve of OFLX solutions was constructed in the concentration range of 0.05–10 mg L^−1^. The limit of quantitation was 0.05 mg L^−1^.

### 4.4. Liquid Chromatography with Mass Spectrometer Detector

Instrumental analysis was performed using a UHPLC Dionex Ultimate 3000 with a Q-Exactive spectrometer, as described previously [38]. Briefly, solvents, acetonitrile hypergrade for LC-MS (LiChrosolv), and formic acid 98% were provided by Merck (Darmstadt, Germany). The instrumental analysis was performed using a UHPLC Dionex Ultimate 3000 system with a Q-Exactive hybrid quadrupole-orbitrap mass spectrometer system (MS/MS) equipped with heat electrospray ionization (HESI), an online vacuum degasser, a quaternary pump, an autosampler, and a thermostated column compartment. The HESI was operated in positive mode. Full MS scans were acquired over the *m*/*z* 70–850 range with a resolution of 70,000 (*m*/*z* 200). The target value was set at 3.00 × 10^6^. Fragmentation was performed in separate runs using a normalized collision energy of 20, 40, and 60 eV. The tandem mass spectrum was acquired with a resolution of 17,500 at *m*/*z* 200. The target value was 5 × 10^4^. Standard mass spectrometric conditions for all experiments were as follows: spray voltage, 3.5 kV; sheath gas pressure: 60 arb; aux gas pressure: 20 arb; sweep gas pressure: 0 arb, heated capillary temperature, 320 °C; loop count: 3; isolation window: *m*/*z* 1.0; and dynamic exclusion: 6.0 s. For all full scan measurements, lock-mass ions from ambient air (*m*/*z* 445.1200 and 291.2842) were used as internal calibrants. All data collected in profile mode were acquired and processed using Thermo Xcalibur 3.0 software and Compound Discoverer 2.1, respectively. Chromatographic separation was achieved with a Kinetex RP-18 column (100 mm × 4.6 mm, 2.6 µm) supplied by Phenomenex (Torrance, CA, USA) equipped with a security guard. The column temperature was maintained at 40 °C with a flow rate of 0.3 mL min^−1^. The mobile phases consisted of HPLC-grade water with 0.1% formic acid as eluent A and acetonitrile with 0.1% formic acid as eluent B. The gradient B (%) was as follows: 0 min—5%; 1 min—5%; 13 min—95%; and 20 min—95%. The re-equilibration of the column to the initial conditions lasted for 5 min. The volume of injection was 10 µL.

Tentative metabolites were detected using Compound Discoverer Software (Thermo Fisher Scientific, Waltham, MA, USA).

### 4.5. Microbial Assay for Risk Assessment

MARA plates and growth media were purchased from NCIMB Ltd. (Aberdeen, UK). The tests were performed according to the standard operational procedure provided by the manufacturer with changes. Initially, the freeze-dried microorganisms were preincubated with 180 µL of the growth medium for 2 h and divided evenly into 3 new 96-well round-bottomed microwell plates. The cultures were diluted 3 times with medium and preincubated for the next 2 h. A 3–fold dilution series of the sample was prepared directly in the plates. Then, the sample serial dilutions were inoculated with the microorganisms in the relevant wells of the plates [22]. The plates were incubated for 18–22 h at 30 °C. The optical density was recorded in the multi-well plate reader (MB-580 HEALES, Shenzhen, China) at 620 nm. The microbial toxic concentration (MTC) for each strain of the microorganism was calculated with MARA software, (https://www.ncimb.com/ecotoxicity-testing/mara-ecotoxicity-test (accessed on 10 March 2025); NCIMB Ltd., Aberdeen, UK) which was provided by the manufacturer.

To show the relative response of each strain in relation to the most sensitive strain, relative sensitivity (RS) values were calculated using the following formula [22]:

RS_i_ = MTC_min_/MTC_i_ * 100, (1)

where RS_i_ is an RS value for the “i” strain; MTC_i_ is the MTC value of the “i” strain, and MTC_min_ is the MTC value of the most sensitive strain.

### 4.6. Microtox^®^

Freeze-dried luminescent bacteria *A. fischeri* and special glass cuvettes were purchased from Modern Water (New Castle, DE, USA). The Microtox^®^ toxicity tests were performed according to the standard operational procedure. Briefly, a series of four 2-fold dilutions of the sample were prepared in the cuvettes. The luminescence of the bacteria incubated in the sample for 15 min was compared with the luminescence of a control sample. The EC50 values were calculated using dedicated software supplied by Modern Water (New Castle, DE, USA).

### 4.7. Spirotox Assay

The Spirotox assay with the ciliate protozoan *S. ambiguum* was performed according to the standard operational procedure, with changes [37,39]. Briefly, the tests were performed in polystyrene 48-well plates, with two replicates per plate. A series of seven 1.5–fold dilutions of the test samples and control were prepared in the multi-well plates. Ten organisms were added to each well containing 0.3 mL of the tested sample. After 24 h incubations at 25 °C in the dark, test effects including morphological deformations and lethality were observed with a dissection microscope (SMZ 1500, Nikon, Tokyo, Japan). Based on the observed percentage of the test effect, the EC_50_ values were determined by using the graphical interpolation method.

### 4.8. Data Treatment

The concentration addition approach was used to calculate the predicted toxicity of the samples. The predicted toxicity unit (PTU) was calculated with the following equation:

PTU = ΣC_i_/Tox_i_, (2)

where C_i_—concentration of the “i” compound in the sample in mg L^−1^; Tox_i_—toxicity value of the “i” compound expressed in mg L^−1^ (MTC for MARA or EC_50_ in case of Spirotox and Microtox^®^).

The measured toxicity unit (MTU) was calculated according to the following equation:

MTU = 100/Tox_i_, (3)

where Tox_i_—toxicity value of the mixture expressed in % (MTC for each MARA strain).

## 5. Conclusions

Photodegradation of OFLX in a sunlight simulator leads to a reduction in antibacterial activity. This has two consequences, one for health, and a second for the environment. From a health point of view, people using OFLX eye drops should not expose the drops or their eyes to sunlight. From an environmental point of view, it is important that OFLX is photodegradable, and its photoproducts are less effective and do not have a different spectrum of bacteriostatic action. This was shown by the unique MARA test using 11 microorganisms with different sensitivities.

Current studies have shown that the photodegradation process of OFLX in minimal medium and in eye drop formulations is very similar. Future studies should evaluate the photodegradation of OFLX dissolved in different surface waters. This will enable the assessment of the influence of substances that both slow down photodegradation (scavengers) and/or accelerate it (e.g., humic acids).

Photodegradation of OFLX leads to the formation of low-toxicity substances with toxic effects on eukaryotic organisms. Hence, in the study of the transformation of antibacterial compounds, it is also necessary to use other bioassays which are not only used for a specific target. Additional organisms should come from different taxonomic groups, which may be related to their reaction to chemical compounds with different structures. Such an action may allow for the capture of other, unexpected test effects, for example, the mutagenicity and induction of the resistance of microorganisms to the bacteriostatic action of compounds.

## Figures and Tables

**Table 1:** The toxicity of the ofloxacin standard solution (OFLX) and 0.3% Floxal eye drops (Floxal). The microbic toxic concentration (MTC) values are expressed as OFLX. The results of both test repetitions (1 and 2) are given.

Microplate Column	Species	Phylogenetic Group	Sample	No ^1^	MTC [μg L^−1^]	MTC [nM]
6	*Delftia acidovorans*	Gram− β-proteobacteria	OFLX	1	7.6	21.0
				2	10.6	29.3
			Floxal	1	21.2	58.6
				2	9.4	26.0
3	*Citrobacter freudii*	Gram− γ-proteobacteria	OFLX	1	75.8	210
				2	68.6	190
			Floxal	1	89.4	247
				2	68.6	190
4	*Comamonas testosteroni*	Gram− α-proteobacteria	OFLX	1	74.1	205
				2	65.9	182
			Floxal	1	109	303
				2	121	334
7	*Kurthia gibsonii*	Gram+	OFLX	1	539	1492
				2	417	1154
			Floxal	1	439	1215
				2	493	1364
1	*Microbacterium sp*	Gram+	OFLX	1	539	1492
				2	605	1675
			Floxal	1	602	1667
				2	271	749
9	*Pseudomonas aurantiaca*	Gram− γ-proteobacteria	OFLX	1	674	1865
				2	484	1340
			Floxal	1	1551	4293
				2	433	1199
8	*Staphylococcus warneri*	Gram+	OFLX	1	NT ^2^	
				2	605	1675
			Floxal	1	NT ^2^	
				2	1065	2947
2	*Brevundimonas diminuta*	Gram− α-proteobacteria	OFLX	1, 2	NT ^2^	
			Floxal	1, 2	NT ^2^	
5	*Enterococcus casseliflavus*	Gram+	OFLX	1, 2	NT ^2^	
			Floxal	1, 2	NT ^2^	
10	*Serratia rubidaea*	Gram− γ-proteobacteria	OFLX		NT ^2^	
			Floxal		NT ^2^	
11	*Pichia anomala*	Yeast	OFLX		NT ^2^	
			Floxal		NT ^2^	

^1^ Number of replicates; ^2^ non-toxic to the highest tested concentration 1500 μg L^−1^.

**Table 2:** The toxicity of the OFLX standard solution (OFLX) and Floxal eye drops (Floxal) during photodegradation in the Spirotox and Microtox^®^ assays. The results of both test repetitions (1 and 2) are given. The EC_50_ values are expressed in % of the solution with an OFLX initial concentration of 30 mg L^−1^ (100% sample).

Irradiation Time [min]	Microtox^®^ EC_50_ [%]				Spirotox EC_50_ [%]			
	OFLX		Floxal		OFLX		Floxal	
	1	2	1	2	1	2	1	2
0	NT ^1^	NT	NT	NT	NT	NT	NT	NT
30	NT	NT	NT	NT	44.5	69.4	70.7	70.7
45	NT	NT	97.4	NT	35.4	35.4	42.6	36.0
60	NT	NT	80.8	NT	32.4	35.4	35.4	34.0
90	97.4	85.1	76.8	70.8	17.7	26.6	33.3	32.4
120	76.1	76.4	67.7	64.8	17.7	29.4	32.4	19.3

^1^—sample is non-toxic, with an effect of less than 50% in the highest tested concentration.

**Table 3:** Tentative identification of degradation products of OFLX and their MS parameters. Identification was performed based on *m*/*z*, isotopic pattern, and fragmentation spectra (coverage by in silico fragmentation).

Name	Formula	Calc. MW ^1^	Δ Mass [ppm]	RT [min]	RDBE ^2^	H/C ^3^	Selected MS/MS Fragments [*m*/*z*]
P391	C_18_H_18_FN_3_O_6_	391.1180	0.06	11.4	11	1.0	86.0599; 237.0308; 279.0772; 374.1139
P363	C_17_H_18_FN_3_O_5_	363.1229	−0.42	4.8	10	1.1	86.0599; 235.0877; 289.0977; 333.0877
P349	C_16_H_16_FN_3_O_5_	349.1071	−0.81	4.8	10	1.0	72.0445; 279.0774; 289.0981; 330.1092
P348	C_17_H_20_N_2_O_6_	348.1316	−1.47	4.7	9	1.2	70.0651; 175.0726; 279.0757
P347	C_17_H_18_FN_3_O_4_	347.1283	0.64	4.9	10	1.1	70.0652; 221.0718; 261.1032; 304.1452
P345	C_17_H_16_FN_3_O_4_	345.1128	0.93	4.9	11	0.9	70.0651; 221.0718; 261.1033; 302.1292
P319b	C_15_H_17_N_3_O_5_	319.1168	−0.10	4.4	9	1.1	218.0917; 244.0715; 262.0817; 276.0967
P319a	C_14_H_13_N_3_O_6_	319.0806	0.60	4.3	10	0.9	70.0287; 72.0443; 143.0238; 161.0688
P306	C_14_H_11_FN_2_O_5_	306.0650	−0.66	10.9	10	0.8	238.0377; 261.0668; 279.0781
P304	C_15_H_13_FN_2_O_4_	304.0860	0.27	4.7	10	0.9	207.0564; 219.0561; 235.0875; 261.1040
P289	C_14_H_15_N_3_O_4_	289.1066	1.01	4.2	9	1.1	58.0654; 145.5602; 202.0971; 233.0556
P283	C_12_H_13_NO_7_	283.0690	−0.61	4.4	7	1.1	58.0654; 180.0291; 208.0241; 226.0344
P278	C_13_H_11_FN_2_O_4_	278.0702	−0.44	12.2	9	0.8	191.0250; 219.0199; 238.0376; 261.0668
P275	C_13_H_13_N_3_O_4_	275.0910	1.39	4.2	9	1.0	118.0286; 138.5522; 189.0657; 233.0550
P267	C_12_H_13_NO_6_	267.0741	−0.68	4.5	7	1.1	59.0494; 182.0449; 192.0296; 210.0398
P263	C_13_H_10_FNO_4_	263.0594	0.00	13.1	9	0.8	202.0671; 224.0339; 246.0558
P255	C_11_H_13_NO_6_	255.0740	−1.10	4.8	6	1.2	180.0290; 220.0603; 210.0754; 238.0707
P249	C_12_H_11_NO_5_	249.0635	−0.77	4.4	8	0.9	149.0601; 164.0341; 192.0288; 232.0602
P225	C_10_H_11_NO_5_	225.0635	−1.16	4.7	6	1.1	59.0493; 108.0444; 152.0342; 210.0758
P128	C_5_H_8_N_2_O_2_	128.0585	−0.55	4.5	3	1.6	111.0554; 85.0761; 58.0655
P119	C_4_H_9_NO_3_	119.0583	0.34	4.5	1	2.2	56.0499; 74.0600; 102.0545
P100	C_5_H_12_N_2_	100.1000	−0.10	4.1	1	2.4	58.0654; 70.0653; 84.0806; 99.0915

^1^ Monoisotopic molecular mass; ^2^ equivalent rings and double bonds; ^3^ hydrogen versus carbon atom ratio.

## Data Availability

Data is contained within the article and Supplementary Material.

## Supplementary Materials

The supporting information can be downloaded at: https://www.mdpi.com/article/10.3390/ijms26062595/s1.
