# Supplementary material for: The Microbial Assay for Risk Assessment (MARA) in the Assessment of the Antimicrobial Activity of Ofloxacin and Its Photoproducts
Source: Int J Mol Sci. 2025 Mar 13;26(6):2595. doi: 10.3390/ijms26062595 (PMC11942207; doi:10.3390/ijms26062595)
Supplement: Supplementary file 1 [file ijms-26-02595-s001.zip › ijms-3462509-supplementary.pdf]

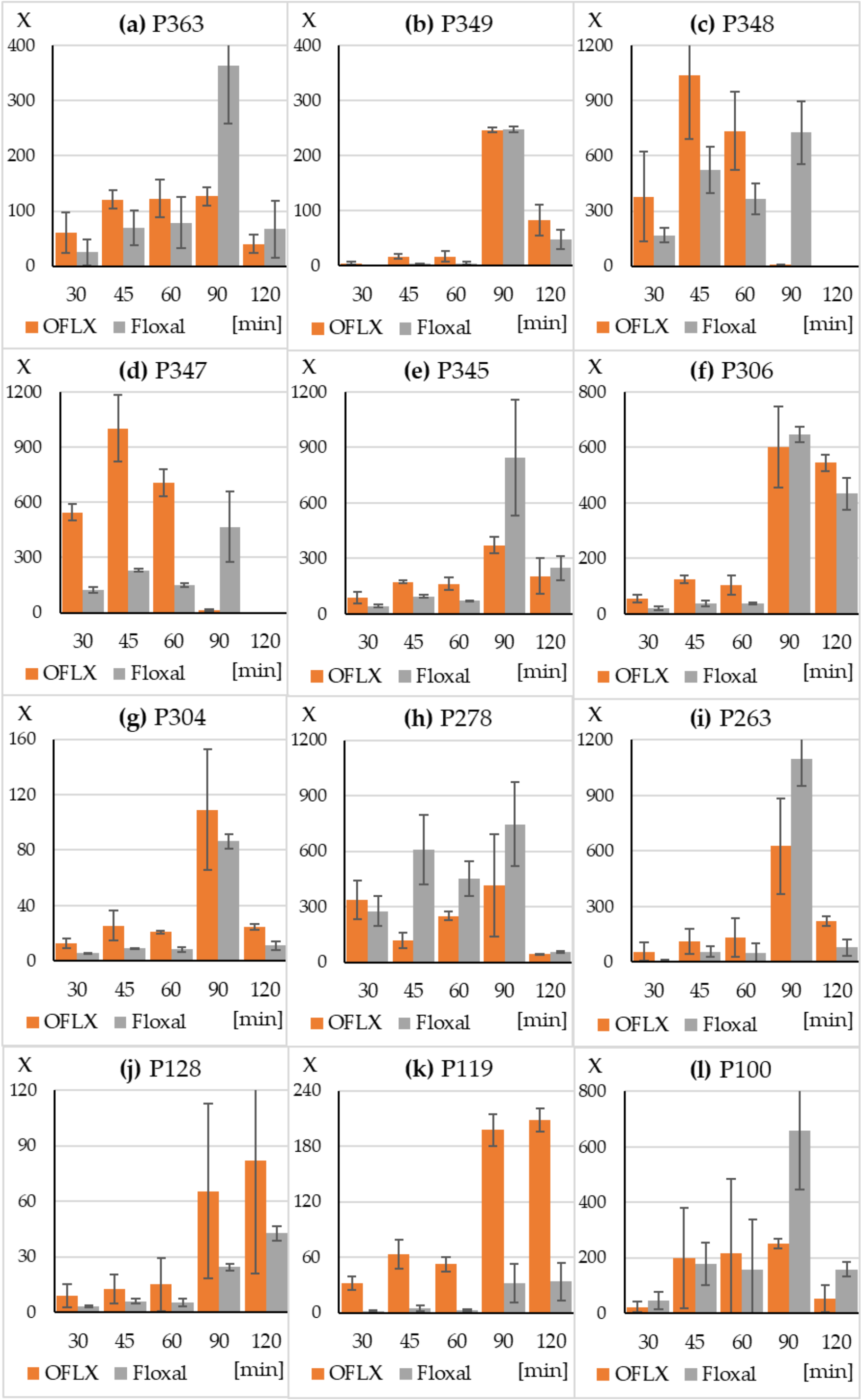

Figure S1. Relative abundance of the OFLX photoproducts during irradiation.

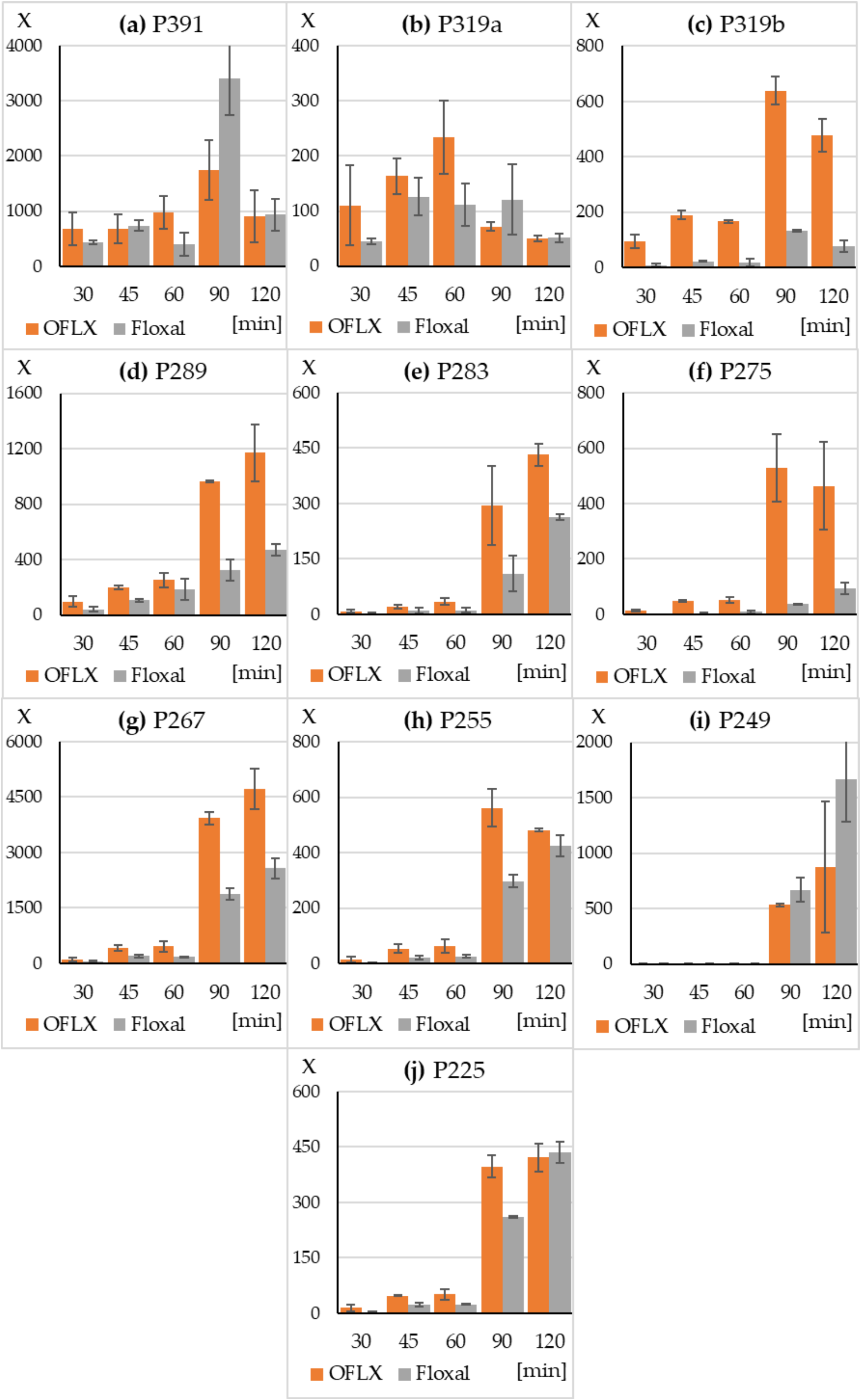

Figure S2. Relative abundance of the OFLX photoproducts during irradiation.
